# Nanomotor tracking experiments at the edge of reproducibility

**DOI:** 10.1038/s41598-019-49527-w

**Published:** 2019-09-13

**Authors:** Filip Novotný, Martin Pumera

**Affiliations:** 1 Center for Advanced Functional Nanorobots, Dept. of Inorganic Chemistry, Faculty of Chemical Technology, University of Chemistry and Technology in Prague, Technická 5, Prague, CZ-166 28 Czech Republic; 2 Future Energy and Innovation Laboratory, Central European Institute of Technology, Brno University of Technology, Purkyňova 656/123, Brno, CZ-616 00 Czech Republic; 3 Department of Chemical and Biomolecular Engineering, Yonsei University, 50 Yonsei-ro, Seodaemun-gu, Seoul, 03722 Korea

**Keywords:** Molecular machines and motors, Molecular machines and motors

## Abstract

The emerging field of self-propelling micro/nanorobots is teeming with a wide variety of novel micro/nanostructures, which are tested here for self-propulsion in a liquid environment. As the size of these microscopic movers diminishes into the fully nanosized region, the ballistic paths of an active micromotor become a random walk of colloidal particles. To test such colloidal samples for self-propulsion, the commonly adopted “golden rule” is to refer to the mean squared displacement (MSD) function of the measured particle tracks. The practical significance of the result strongly depends on the amount of collected particle data and the sampling rate of the particle track. Because micro/nanomotor preparation methods are mostly low-yield, the amount of used experimental data in published results is often on the edge of reproducibility. To address the situation, we perform MSD analysis on an experimental as well as simulated dataset. These data are used to explore the effects of MSD analysis on limited data and several situations where the lack of data can lead to insignificant results.

## Introduction

The field of self-propelled nanorobots has already attracted tremendous interest^1–3^. These autonomous nano-sized machines, which can navigate the environment and act on external signals, have many promising applications^2^. Starting with micromotors^1^, which can be tracked and visualized *in situ* at standard atmospheric conditions using a conventional wide-field optical microscope^4,5^, the nanorobots field has now achieved robot sizes in hundreds of nanometers^6–8^. At such sizes, the optical microscopy diffraction limit and the decrease in visibility degrades the ability to track motors with dimensions below 300 nm while maintaining the information of shape. The increasing dominance of Brownian forces turns the apparent ballistic paths of micromotors to random walks of colloidal particles^6^. Despite the challenges, the potential applications of smaller (i.e., fully nanosized) are very tempting^9^. Such small nanorobots would be able to navigate living tissue and penetrate the cell environment without alerting the organism’s immune response as do larger, micro-sized objects^10^. Recent works consider nanosized nanomotors and their applications^6–8,11^; however, the detection and tracking methods need to be adapted to the smaller sizes^12–14^. Optical setups allow localizing a nanoparticle even if its size is below the diffraction limit to scatter incident light^13,15–18^. The particle would then appear as a radiating point, which in some approximation corresponds with the nanoparticle’s location^14,19,20^.

Recent nanomotor literature has not yet reached agreement on the complete experimental information about the procedure used to evaluate the enhanced diffusion of the colloidal motors using the tracking of a nanoparticle’s path. Most of the articles in the field use the mean squared displacement (MSD) function and the number of analyzed tracks per sample is often in the order of tens^6–8^. Although much has been done in the literature on the diffusion of sub-micron particles in complex environments and the analysis of the diffusive processes through statistical analysis of tracked particle paths^21–24^, number of experimental works lacks the rigorous perspective.

Despite the extensive coverage of the topic, we feel the need to address the challenges of the particle tracking analysis specifically when applied to the evaluation of propelled motion of catalytic “motors”, whose sizes lie at the threshold between sedimenting microparticles and true colloidal nanoparticles. Therefore, we demonstrate the types of errors and misinterpretation of nanoparticle tracking data in the context of self-propulsion of colloidal particles while using the MSD function. Unlike previous articles discussing the matter on a set of designed physical experiments^13^, we approach the discussion using generated random-walk data. To achieve the best clarity, we generate three-dimensional Brownian particle tracks using a random process generator and then analyze the data using the methodology used in the majority of nanorobot papers. To give a real-world example, we also include the particle tracking data of size-standard 100 nm polystyrene spheres in water and show that depending on the dataset size and whether we “hand-pick” the particles, one can “prove” by MSD analysis on such a small dataset that these particles are self-propelled, even though they are not.

## Results and Discussion

In this paper, we specifically approach the matter of tracking the motion of self-propelled nanorobots and the data analysis in the manner of recent nanorobot articles to show the possible misinterpretations of the MSD method. We perform MSD analysis on an experimental as well as simulated dataset. We first show tracking and data analyses of the experimentally recorded movement of 100 nm latex beads with low size dispersion, where we demonstrate that depending on the dataset and its size, one can mistakenly “prove” that these latex nanoparticles exhibit enhanced diffusion. Subsequently, we provide an analysis on numerically simulated tracks to gain insight into the method and to propose the limits of the method to analyze nanorobot motion in the sense of enhanced diffusion. In the scope of the methodology of nanorobot papers, we answer the question of what the dataset size is needed to provide statistically practical information.

First, we analyze the movement of the size-standard 100 nm latex particles (LPs) using a dedicated nanoparticle tracking instrument Nanosight NS300 (Malvern). The industry standard analysis yields the size distribution of the measured particles based on tracking multiple particles in a temperature-stabilized controlled optical geometry and then analyzing the particle tracks using the MSD function. At its most straightforward implementation, the Einstein–Stokes theory predicts the MSD function to rise linearly in time and that the relations between the mean squared displacement 〈*L*^2^〉, the diameter of the diffusing particle *d*, and its diffusion coefficient *D* are as follows:

1 $$\langle {L}^{2}\rangle =2{\epsilon }Dt,\,\,D=\frac{{k}_{B}T}{3\pi {\eta }_{0}d},$$

where *ϵ* is the number of axes of freedom of the particle movement, *k*_*B*_ is the Boltzmann constant, *T* is the absolute temperature, *η*_0_ is the viscosity of the medium in which the particles are dispersed, and *d* is the diameter of the particle. The MSD analysis consists of gathering the squared displacements at a specific time difference 〈*L*^2^〉(Δ*t*) from all the measured particle tracks. The squared displacements gathered by the time difference are used to construct an MSD function from all the track data.

**Figure 1:**
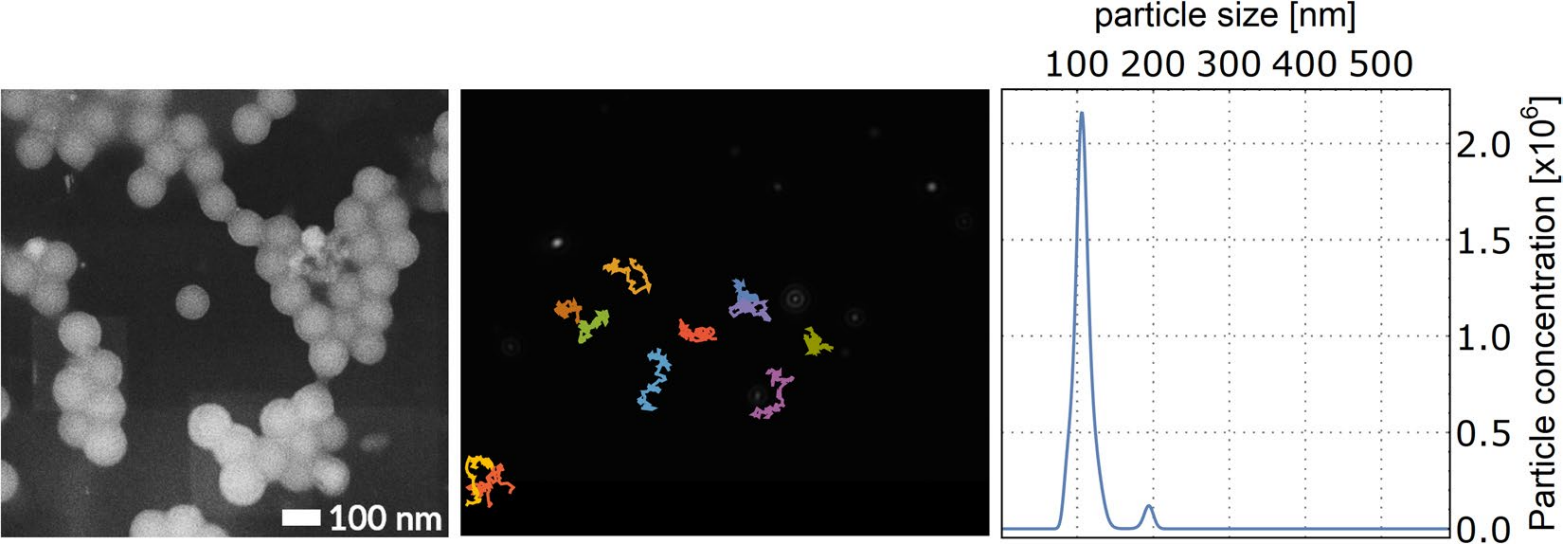
Basic characterization of the used 100 nm latex size-standard sample. (Left) FEG-SEM sample of the drop-casted 100 nm latex sphere colloid. (Middle) Single frame from the .avi video file taken by Nanosight NS300 instrument with the 100 nm latex spheres. Overlaid are the measured particle tracks picked for the visualizations in this work. (Right) 100 nm latex size-standard sample size distribution as analyzed by the NTA 3.2 software (Malvern). The median particle size derived by the NTA 3.0 software (FTLA method) is 104.3 nm and the corresponding experimentally derived diffusion coefficient is 470.8 × 10^4^ nm^2^/s.

As an example, the MSD analysis of a 60-second capture of Brownian movement of the LPs selected in our work yields the diffusion coefficient and from that the software derives the particle size. We present the processed output of the NTA done on the 100 nm size-standard sample in Fig. 1, together with the SEM image. Using a refined finite track length adjustment (FTLA) algorithm^15^, the NTA 3.0 software outputs the mean of the primary peak of the sample size distribution to be 104.3 nm, a results well in accordance with the declared particle size and the specifications of the NS300 instrument. The intermediate diffusion coefficient distribution has main peak at 469.6 × 10^4^ nm^2^/s, as derived from the Eq. (1) (the exact theoretical diffusion constant of 104.3 nm spherical particle in water at 25 °C equals 470.8 × 10^4^ nm^2^/s).

We use the NS300 setup to explore the intermediate result of the method and raw particle tracks and focus on the MSD analysis as used in micro/nanomotors papers. The quality of the nanoparticle tracking (NTA) setup (temperature stabilization, low dark-field imaging background) allows us to focus on the details of the MSD analysis instead of discussing the setup itself. During the 60-second video capture at 25 frames per second (FPS), a total of 244 valid particle tracks were acquired (see Fig. 1: center for representation of a few selected tracks of particles present in the showed video-frame). We exported the raw particle tracks and performed an MSD analysis of the tracks to derive the mean diffusion coefficient of the ensemble.

In experimental particle tracks, additional noise is present as a result of the error of determining particle position due to limited resolution and further effect is introduced by blurring the position due to the non-zero exposure time for each video frame^14,20^. Those uncertainties can be addressed by adding a constant term in the relation between the MSD and the diffusion coefficient *D*^15,22,25^. In our case, however, the methodology of the nanomotor papers we are addressing is using the simplest case, fitting the MSD function with the equation:

2 $$\langle \Delta {L}^{2}\rangle =4D\Delta t,$$

which yields the desired diffusion coefficient *D*^6,8^.

**Figure 2:**
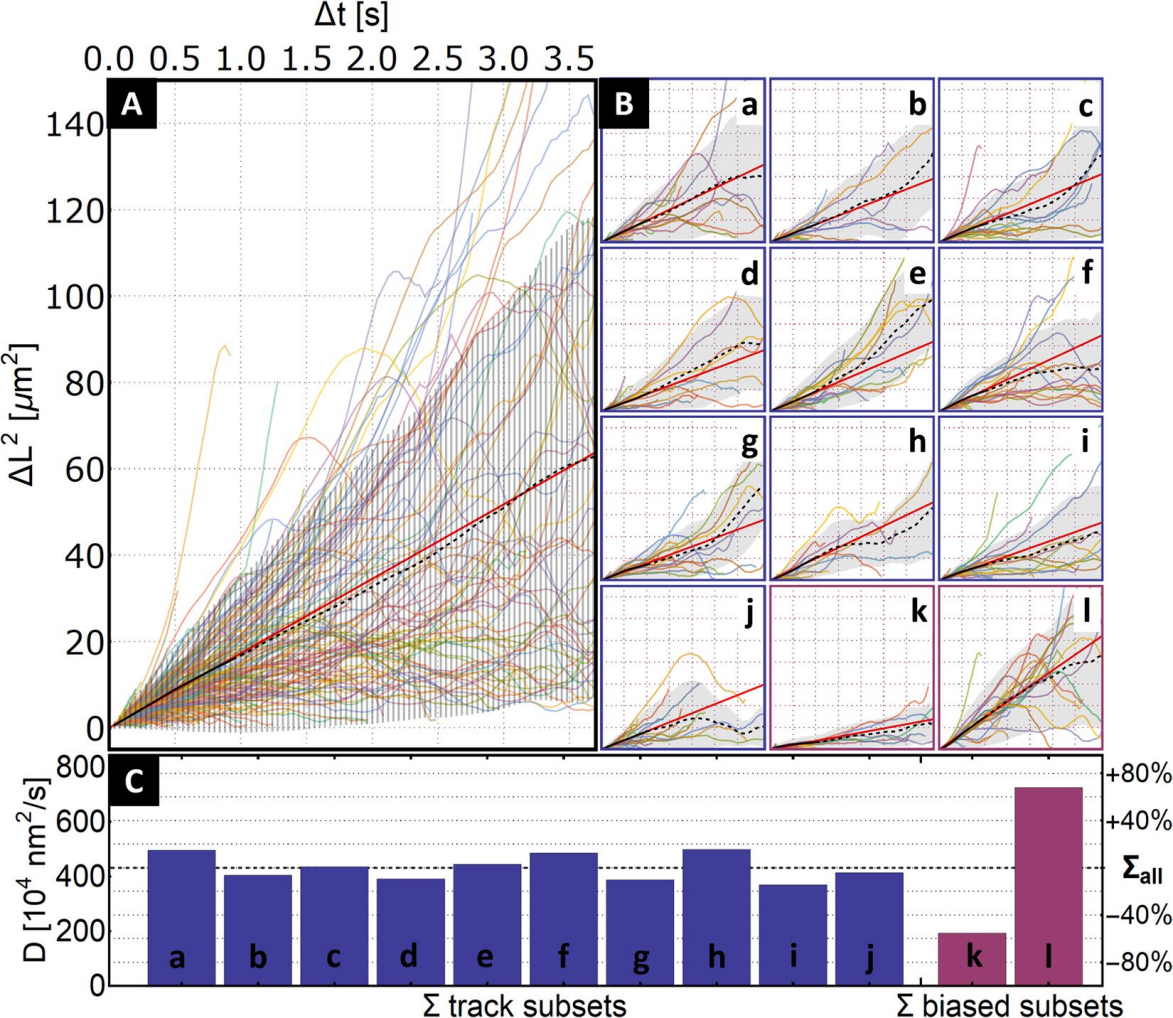
Demonstration of the spread of diffusion coefficients derived from the MSD analysis on experimental 100 nm Latex sphere sample. (**A**) Plot of MSD functions of all 244 measured 100 nm latex particle tracks (in colors) with the averaged MSD function derived from the whole particle assembly (black). The red line represents the fit of the first one-fourth of Δt values by the Eq. (1). (**B**) The subplots a–j (blue) correspond to MSD analyses of a subset of 24 tracks of the 100 nm latex particle tracks. The subplots k, l (purple) correspond to biased selection of 24 tracks from the measured particle set based on the lowest respective highest diffusion. (**C**) Resulting diffusion coefficients from the MSD analysis. The indicated ∑_all_ value (431.6 × 10^4^ nm^2^/s) is a diffusion coefficient derived by MSD analysis of the whole track ensemble. Blue bars a–j are values of diffusion coefficients corresponding to the respective subplots a–j in B. The purple bars k, l are diffusion coefficient values corresponding to the MSD analysis of the biased selection of tracks from subplots k, l. The low number of particle tracks analyzed provides ±30% deviation in the derived diffusion coefficient. As an extreme situation, the two biased subsets that were provided change the results, respectively, to −55% and +65% with respect to the value derived from the analysis of the whole experimental dataset of 244 tracks.

The plot of the individual MSD functions for each tracked LP, the averaged MSD, and the respective fitted linear function from the whole measured ensemble of LPs are shown in Fig. 2A. Next, we analyze what happens when we limit the experimental data further to a few tens of tracks for an analyzed sample. We partition the set of 244 particle paths into groups of 24 particles and repeat the MSD analysis. The resulting plots of the MSD analysis of the tracks’ subsets are shown in Fig. 2B together with the respective values of their diffusion coefficients in Fig. 2C.

Doing the MSD analysis of those subsets proves the problems with scarce data in any statistical method^26^: MSD analysis on such limited data yields a significant spread in the derived diffusion coefficient value. Namely, when selecting the subsets with minimal and maximal diffusion coefficients, we get values 369.077 × 10^4^ nm^2^/s and 498.002 × 10^4^ nm^2^/s, corresponding to a ±30% change in the diffusion coefficient acquired from the analysis of the whole sample. When we consider the output of the biased set, the situation is expectedly worse. The diffusion coefficients equal to 192.122 × 10^4^ nm^2^/s and 724.761 × 10^4^ nm^2^/s, resulting, respectively, in a −55.5% and +68.0% change from the diffusion coefficient of the whole ensemble.

The striking difference in the resulting diffusion coefficients illustrates the point we are addressing: when one can include only a limited number of particle tracks, the resulting MSDS analysis differs among the different subsets even if the particles belong to the same sample with minimal physical properties (size-standard 100 nm latex spheres)^27^. Including only 24 particle tracks, we get the spread of the resulting diffusion coefficients over the sample, where the highest is changed by 86% in respect to the lowest. When using the biased selection, the ratio between the results is near 3.8x. The difference in the diffusion coefficient - more precisely the apparent increase of the diffusion coefficient - can be interpreted as an enhanced diffusion, which suggests that the particles possess some amount of self-propulsion. Moreover, there is inherent cause in the single particle tracking method, which can be falsely identified as an inhomogeneity in the sample diffusion, which is even more enhanced when using scarce data^27^.

### Constructing the simulated dataset of pure Brownian movers

Consequently, we move to provide a peek at the deep underlying reasons for the observed “skewed” results and to answer the question on the proper method to analyze the motion of these nanoparticles. To gain insight into the problem, we generated a set of 1,000 three-dimensional random walks simulating the Brownian movement of particles. Assuming the size, we neglect any contribution of inertia in the particle movement, which simplifies the problem. We normalized the simulated tracks by a factor $k=\sqrt{2{D}_{th}\Delta t}$, where *D*_*th*_ is the theoretical diffusion coefficient of a 104.3 nm sphere at our experimental conditions and Δ*t* is the frame length used in our experimental setup, 0.04 second. By the multiplication of the factor *k*, the simulated paths resemble the 104.3 nm size spherical particles as measured by the NTA 3.0 software used in the Nanosight instrument. The track ensemble was then tested by MSD analysis, where it yielded a diffusion coefficient for the selected size of particles in good agreement with the theoretical *D*_*th*_ (see ESI, Fig. S1). Such a simulated dataset has the advantage that we can directly compare the results of changing analytical parameters without introducing any more uncertainties by using different data for each experiment. We did this to gain insight into the MSD analysis and the limitations one should consider when analyzing a sample of colloidal particles for self-propulsion.

There are several key experimental issues with tracking nanorobots. We want to address those that arise during the transition from micromotors to fully colloidal nanomotors. Namely, using the standard optical microscope, we always reduce the three-dimensional pathways of a colloidal particle to a two-dimensional projection. To derive the actual nanopropulsion speed, one needs to achieve FPS at a sufficiently high enough for the video frame time length to be significantly lower than the rotational diffusion relaxation time constant of the nanorobots^24^. For a 100 nm colloidal sphere, this equals 776 milliseconds and approximately 15,000 FPS. We will explicitly address these situations in the chapters below.

### Effect of reducing a three-dimensional path to a two-dimensional projection

**Figure 3:**
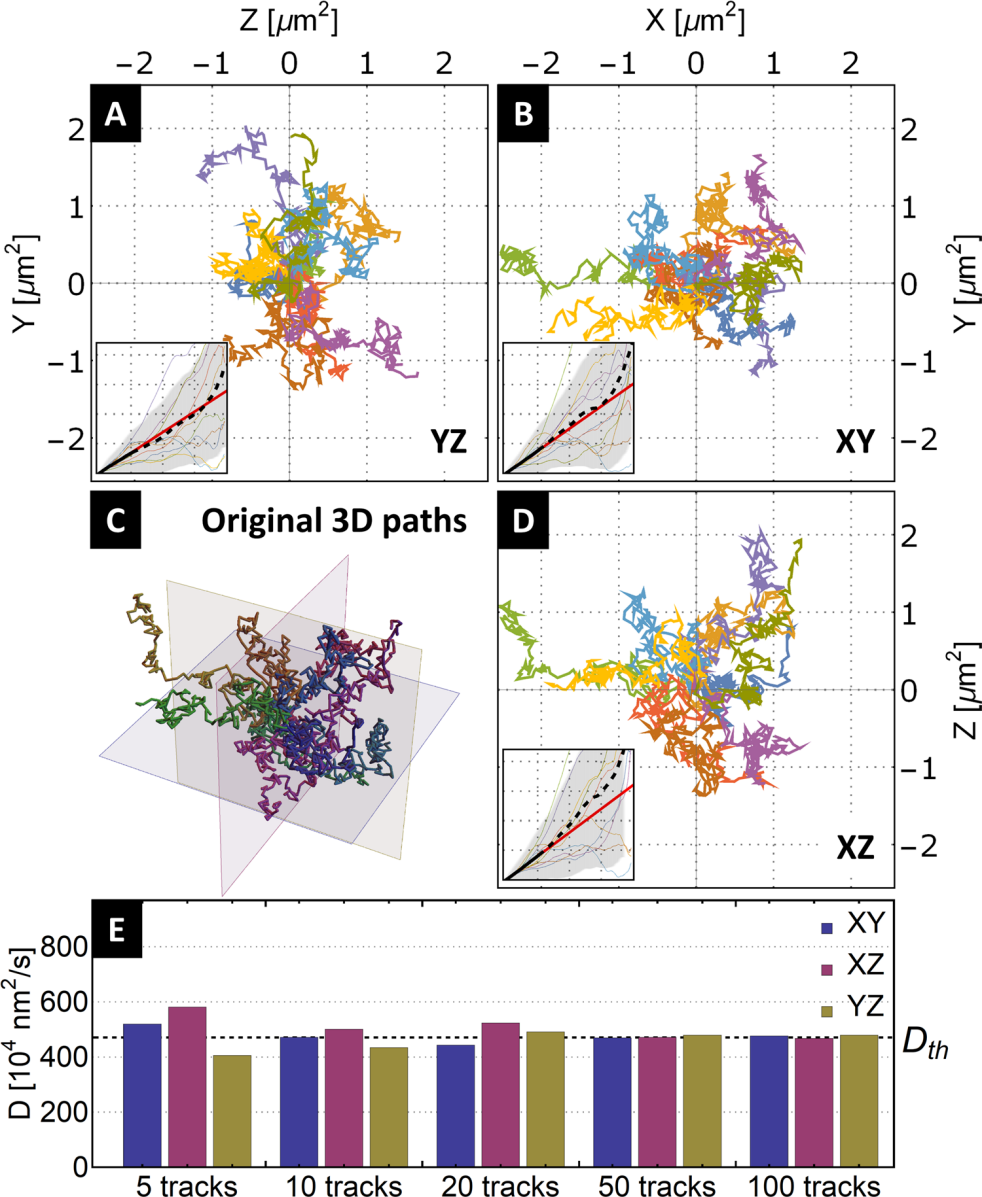
Effect of projecting the three-dimensional Brownian paths to a set of three orthogonal two-dimensional planes. (**A,B,D**) Projections of a set of 10 three-dimensional Brownian paths to three orthogonal planes XY, XZ, and YZ. Insets in the two-dimensional projections are the calculated MSDs (color) together with averaged MSDs (black) for each track and projection. (**C**) Original 3D Brownian paths. (**E**) Result of MSD analysis yielding the diffusion coefficient for the separate projections and increasing the number of original 3D tracks. The values of diffusion coefficient D differ concerning the selected 2D projections of a small number of analyzed tracks (5, 10, 20). For an increasing number of tracks used for MSD analysis, the value of the diffusion coefficient converges to a common value.

The majority of motor-tracking microscope setups use a set focus plane during the acquisition of particle paths^14^. When considering non-sedimenting colloidal motors, the setup can be viewed as a projection of a three-dimensional path to a two-dimensional plane (objective focus plane). In the standard notation of a three-dimensional space (X, Y, Z), let us explore the results of projecting the simulated Brownian paths to three orthogonal planes XY, XZ, and YZ. For illustration, we visualize 10 of the simulated 3D Brownian paths and their respective 2D projections in Fig. 3A–D.

As can be seen on the two-dimensional projections and their respective MSD analyses (subplots Fig. 3A,B,D), just the act of projecting the three-dimensional path to a two-dimensional plane creates a difference in the derived diffusion coefficient. The difference in the analysis should be mitigated as the number/tracking length of analyzed tracks in the sample increases. In Fig. 3E, we explore this problem by repeating the analysis for an increasing number of tracks. The satisfactory number of tracks and their lengths practically depend on a specific situation. In our case, we find that using at least 50 tracks from the simulated set keeps the spread in the resulting diffusion coefficient, caused by the projection of three-dimensional paths to a two-dimensional plane, within the practical limits of experimental measurement.

**Figure 4:**
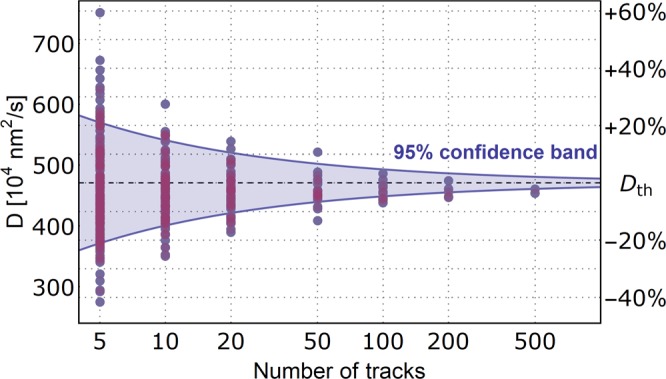
Plot of XY projection MSD analysis results for non-overlapping subsets of the simulated 1,000 three-dimensional track ensemble. The depicted value D_th_ corresponds to the theoretical value of the diffusion coefficient of the 104.3 nm nanoparticle. Overlaid over the point plot is the 95% confidence band derived from published analytical solution based on the total amount of recorded positions and the fraction of the track length used as the maximum Δ*t*^23^.

We follow up the results and use the whole 1,000 track dataset and divide it into non-overlapping subsets of 5, 10, 20, 50, 100, 200, and 500 two-dimensional tracks made by the projection to the XY plane. Next, we compute the diffusion coefficient by MSD analysis of those subsets and plot the results together with the theoretical diffusion coefficient for the selected nanoparticle size on Fig. 4.

The resulting plot gives even more stress on the number of analyzed tracks per sample. Depending on the severity of one’s result, we need to be aware that the variation of the diffusion constant yielded by MSD analysis can be still ±10% at 50 analyzed tracks. The plotted datapoints fits well into the published derivation of upper and lower bounds of the deviation in the MSD analysis^23^ based on the number of sub-step measurements and the total length of the tracks (Fig. 4 - 95% confidence band).

### Interpretation of the MSD trend for the confirmation of nanopropulsion

We consider the would-be nanopropulsion of small colloidal nanoparticles. The rotational correlation time ${\tau }_{C}=4\pi {d}^{3}{\eta }_{0}/3{k}_{B}T$ of such small particles is in the order of microseconds. To be able to assess the trend of the MSD in such case, one has to be able to capture the movement of the particles in much shorter time intervals, say one order of magnitude shorter^13,21^. An upwards curving plot at the very short Δ*t*s indicates propulsion independent of the Brownian movement, whereas a linear trend indicates a natural Brownian movement^13,21^. As for the theoretical exploration of the Brownian motion and self-propulsion, the condition of the sampling interval being much smaller than the rotational diffusion is connected to the fact that in such a case, one can neglect parts of the equation, simplifying it to a form $\langle \Delta {L}^{2}\rangle =4D\Delta t$ ^13,21^. The limiting form is then fitted to the MSD curve; our approach gives an immediate perspective from the experimental measurement part of a problem.

**Figure 5:**
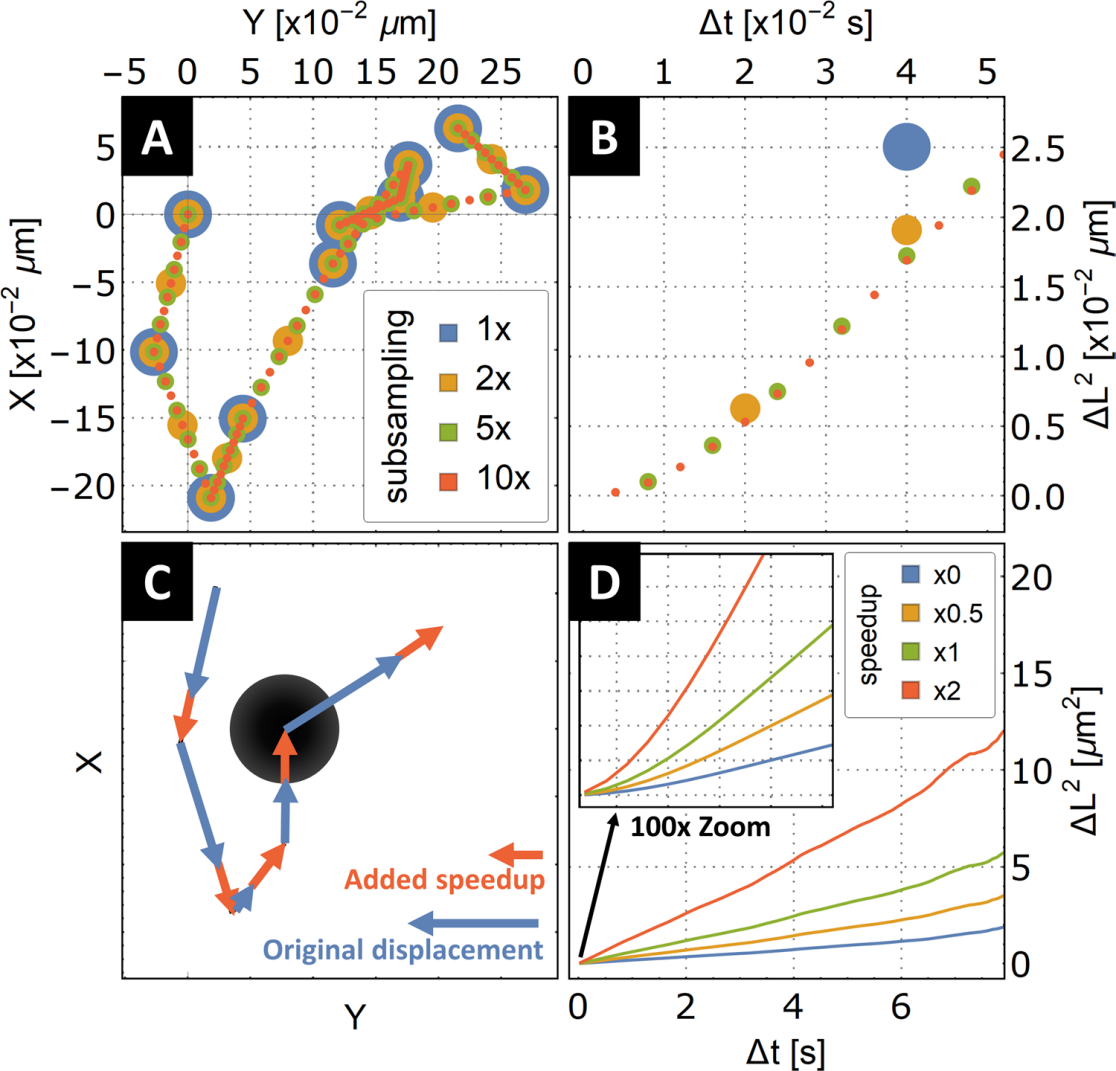
Effect of subsampling the simulated self-propelled particle trajectory, demonstrating the amount of subsampling needed to unravel the proposed parabolic trend at the very beginning of the averaged MSD curve used to determine propulsion speed. (**A**) Small segment of one simulated self-propelled particle track and visualization of the subsampling of the initial simulated particle location. (**B**) MSD analysis done on the self-propelled particle track in sub-image A. Depending on the amount of subsampling, the parabolic trend of the beginning of the MSD function appears. Subsampling by order of magnitude (10x) gives confidence in the parabolic trend. (**C**) Schematic depiction of the simulated self-propelled particle. The original particle displacements are augmented by a constant-length step in the direction of the respective displacement. (**D**) Effect of an increasing amount of particle self-propulsion demonstrated on the MSD analysis of sets of 10 simulated particle paths with x0, x0.5, x,1 and x2 added speedup, and x10 subsampling. Here, we demonstrate that considering most of the averaged MSD function, the self-propulsion exhibits itself as an enhanced diffusion, effectively increasing the MSD slope. Only when looking at the very beginning of the MSD function (displacements at very short time differences), can one see the parabolic behavior.

To narrow the vague statement that Δ*t* must be much shorter than the rotational correlation time, on Fig. 4 we explore the effects of propulsion on the simulated particle tracks used in this work. The simulated tracks are modified to include a constant travel “kick” in the direction of the respective movement at the set time interval (Fig. 5C). Using this modification of the artificial Brownian paths, we can now ask the question of how much finer the time sampling of the path must be to see the upward curving of the MSD of such tracks. Figure 5 explores this question in detail. The respective subsampling of a small segment of the original particle track is visualized in Fig. 5A. Figure 5B plots the MSD functions for the respective subsampling of 10 simulated Brownian paths from our 2D projection. We start to see the initial parabolic uptrend of the MSD curve at the 5x subsampling, while 10x subsampling improves confidence in the parabolic trend.

To connect the derived need of subsampling to a real-world example, consider a micromotor approximated as a sphere of diameter 1 µm; then, the rotational correlation time *τ*_*C*_ equals 0.679 seconds. To be able to capture the initial parabolic trend of the MSD in order to asses if the micromotor exhibit directed motion superimposed on its natural Brownian movement, one must be able to sample the particle movement order of magnitude lower than the *τ*_*C*_, which in this situation is roughly 15 FPS $(1/0.679\ast 10\approx 14.73)$. Such speed is readily achievable by a standard camera setup. In the case of a 100 nm nanomotor, the *τ*_*C*_ scales proportionally with *d*^3^; one order of magnitude change in diameter results in a three-order magnitude change in the FPS needed. The framerate needed to analyze the self-propelling velocity in the case of 100 nm nanomotors is 15,000 FPS. This needs a specialized high-speed camera and microscope setup with a bright light source.

### MSD analysis in the condition of framerate comparable of *τ*_*c*_

**Figure 6:**
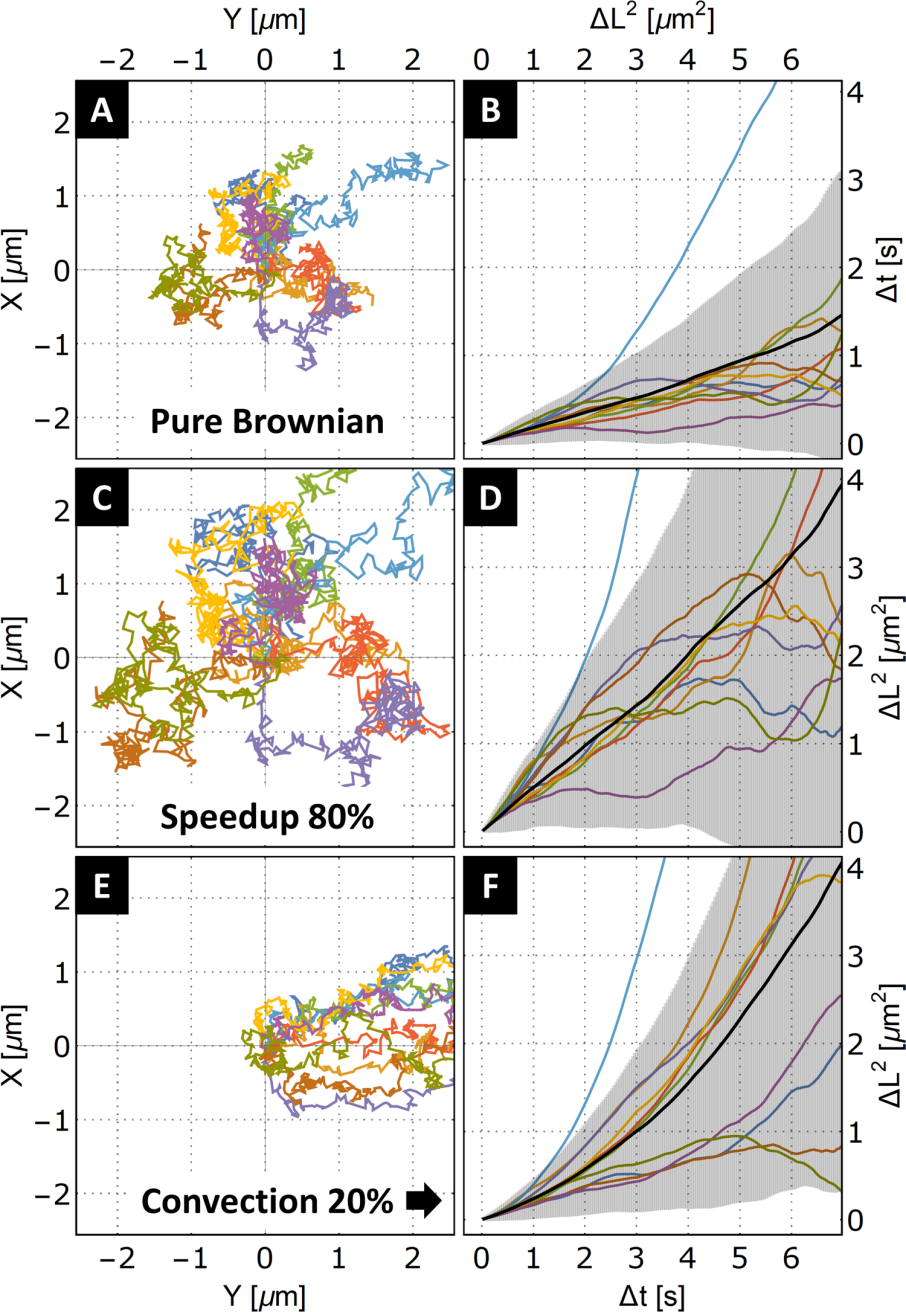
Demonstration of the effect of self-propulsion and convection to an MSD analysis on the same set of 10 two-dimensional tracks. (**A**) Visualization of the original 10 Brownian particle tracks. (**B**) Plot of the individual MSD functions together with the averaged MSD from the whole ensemble of Brownian tracks. (**C**) Visualization of the 10 original Brownian tracks, where a constant speedup was added to the particle displacements, simulating self-propulsion. (**D**) Result of the added propulsion on the MSD analysis. In this data representation, an increased slope of the averaged MSD is apparent. The increased slope can be interpreted as enhanced diffusion. (**E**) Visualization of the 10 original Brownian tracks where a constant directional shift was added to the particle displacements, simulating collective motion such as convection of the medium. (**F**) Result of the added collective motion on the MSD analysis. In this data representation, an upward tilting slope is apparent. The classic method of deriving the diffusion coefficient by fitting with the linear function is not possible in this scenario.

Our simulated tracks provide simple insight into the problem of capturing the particle movement in between the redirection by tumbling. The positions along the simulated trajectory also represent the events of tumbling. Doing the MSD analysis without the subsampling is comparable to running a tracking experiment with framerate compared with rotational correlation time. At such conditions, MSD analysis cannot provide information about the particle velocity and one only obtains information on particle diffusivity. In Fig. 6, we explore the effects of both the individual propulsion of each particle in similar manner as in previous section and of the collectively added movement, which can be caused, for example, by convection in the medium. Analyzing the MSD at time intervals similar to the rotational correlation time, one can only confirm the existence of enhanced diffusion by the increase of the overall MSD slope as seen on Fig. 6B when compared with Fig. 6D.

The collective unidirectional particle movement projects itself in the MSD at longer Δ*t* intervals, curving the trend upward. The simple MSD analysis of fitting by linear function cannot be applied in such a case. On the other hand, such effects are seen at longer time intervals than the rotational diffusion and are, therefore, readily achievable. When capturing the positions of several particles simultaneously, convection can also be directly identified as a joint movement of all the captured particles. This is usually derived from the center of mass of all the particle movements centered around zero^28^. Therefore, in experiments where multiple particles paths are recorded at once (which is often the case in nanoparticle tracking setups), the collective effects can easily be subtracted from the particle paths and mitigated. In some nanomotor cases, however, the individual motors are tracked separately, and the MSD analysis provides invaluable information on the presence of convection or sedimentation.

### Relations between the number of analyzed tracks and the maximum analyzed Δ*t*

Another factor of the MSD analysis not often mentioned in nanorobots papers is the appropriate maximal Δ*t* to which to fit the theoretical trend of MSD. For the results to be coherent across various experiments and to allow for the reproduction of the results, this parameter is of the same importance as the number of tracks. Let us mention two papers where the suggestions are to take Δ*t* up to one-fourth of the duration of the track^29^ and a more recent work suggesting to take up to one-tenth of the track duration^13^. The reasoning behind this reflects the fact that with increasing Δ*t*, the MSD is constructed from a lower number of respective track segments. The decrease in the number of measurements increases the amount of data points in a sub-step which increases the overlap in the data and thus lower the independence of the measurements. This leads in increase in the variance of the derived MSD value at respective Δ*t*^23^. In our work, we confirm that the better result for the MSD analysis of the ensemble is to first take one-fourth of *Δt* from the averaged MSD. Figure S1 provides detailed contour plots showing the combination of the number of tracks and the fraction of the total track length used as maximum Δ*t* needed to achieve significance at certain levels of change in the derived diffusion coefficient.

To increase the number of the subtracted samples at certain Δ*t*, one can either increase the time during which the particle is tracked, or one can track several particles (preferably simultaneously) that follow the same physical behavior. Either option will increase the number of the available subtracts for analysis at specific Δ*t* and increase confidence in the MSD value at this Δ*t*^26^. In nanomotor experimental works, however, we experience the opposite approach: it is difficult to track one particle for extended times (limited field-of-view, limited depth-of-focus) and, in many cases, it is also difficult to accumulate a reasonable number of individual particle tracks^6,7^.

Table 1 provides a general rule-of-thumb for the number of collected tracks needed to ensure statistically significant proof of a certain amount of relative change of derived diffusion coefficient. For example, it demonstrated that to obtain a significant statistical proof that the one observes 10% (or lower) enhancement in diffusion of small Brownian nanomotors, one must collect at least 20 tracks to have significance in 95% confidence or at least 40 tracks to have significance at 99% confidence.

**Table 1:** A rule-of-thumb table for the values of the least number of tracks needed to confirm a change (5%, 10%, and 20%) in observed diffusion coefficient *D* using 25% of the total track duration as the maximum Δ*t* in the analysis.

Lowest significant change of *D*	Tracks to achieve 95% confidence	Tracks to achieve 99% confidence
5%	>90 tracks	>150 tracks
10%	>20 tracks	>40 tracks
20%	>5 tracks	>10 tracks

Table contains values both with 95% confidence and 99% confidence.

## Conclusion

We have demonstrated the sensitivity of MSD analysis of colloidal movers using only a limited number of tracks (<100) for each sample. The primary tool used was a generated dataset of 1,000 simulated paths of pure Brownian characteristics. Using the dataset, we explored the effect of projection of the three-dimensional paths to a two-dimensional plane, simulating the effect of observation of unconstrained colloidal particles in an optical microscope. Subsequently, we modified the dataset to simulate self-propulsion and collective behavior similar to convection. We used the MSD analysis on the modified dataset and plotted the results. Self-propulsion also leads to the question of the estimation of the speed of propulsion. We did a phenomenological exploration of the subsampling of the particle path to be able to fit the data with the model, which involves particle speed. All the demonstrations showed that on average, when below 50 tracks for each studied sample, the MSD analysis becomes unreliable when the goal is to support claims of the enhancement in diffusion by 20% or less.

Even in a pure Brownian system of size-monodispersed latex nanospheres and water, the MSD functions of single-tracked particles present complex behavior apart from the theoretical linear dependence given by the Einstein equations^27^. The real MSD has to be fitted with the presumed equation of expected behavior and a reference model. To increase the strength of the information, one has to assess several hundred particle paths to obtain robust statistics^26^. This must be taken into account if the field of nanorobots is to progress.

## Methods

### Characterization of the 100 nm latex spheres

The movement of the 100 nm size-standard latex spheres (provided by Malvern as a calibration standard) in water was recorded by the Nanosight NS300 nanoparticle tracking system from Malvern Instruments. The device was configured with a 532 nm laser diode and sCMOS camera and 60x optical objective. The provided calibration of the pixel size is 141 nanometers. The sample of latex spheres was diluted by Milli-Q water and injected in the general Nanosight cell for analysis. The NTA 3.2 software was used to do the particle tracking using the gain 6 and detection threshold 30. The detected particle tracks were then exported into an MS Excel file format for the analysis in this paper. To further characterize the latex sphere sample, the NTA 3.2 software was used to estimate the size distribution of the particles using the FTLA method, yielding a mean size of 104.3 nm and diffusion coefficient 470.8 × 10^4^ nm^2^/s. The scanning electron microscope images of the latex particles were acquired by a scanning electron microscope with an FEG electron source (Tescan MAIA3 Triglav™) in immersion mode and using a 5-kV electron beam.

### Generation of simulated 100 nm Brownian particle tracks

A set of 1,000 paths of three-dimensional Brownian movers was generated by the standardized Wiener process for random number generation with Gaussian distribution and using Wolfram Mathematica computation software. The paths were generated 201 steps long; each new step meant an independent addition of a random vector to the previous particle position. All tracks start at coordinates{0,0,0}. We use this first-order approximation of a real Brownian particle. For nanomotors in the order of hundreds of nanometers of a characteristic size, the Reynold number is already small enough that the effects of inertia can be neglected (see Fig. 6A for the visualization of the randomly picked 10 paths from the dataset). Furthermore, to give a zero-order simulation of the effects of nanopropulsion and convection, we introduce a constant shift in the particle position at each generated time step, where the value of the added shift is derived from the mean step size of from all the particles tracks multiplied by factor of 0.8. Figures 6C,E visualize the effects of those modifications on the particle tracks. The shift is added in the direction of the actual displacement for nanopropulsion or in a constant direction for convection.

### Mean squared displacement function algorithm

**Figure 7:**
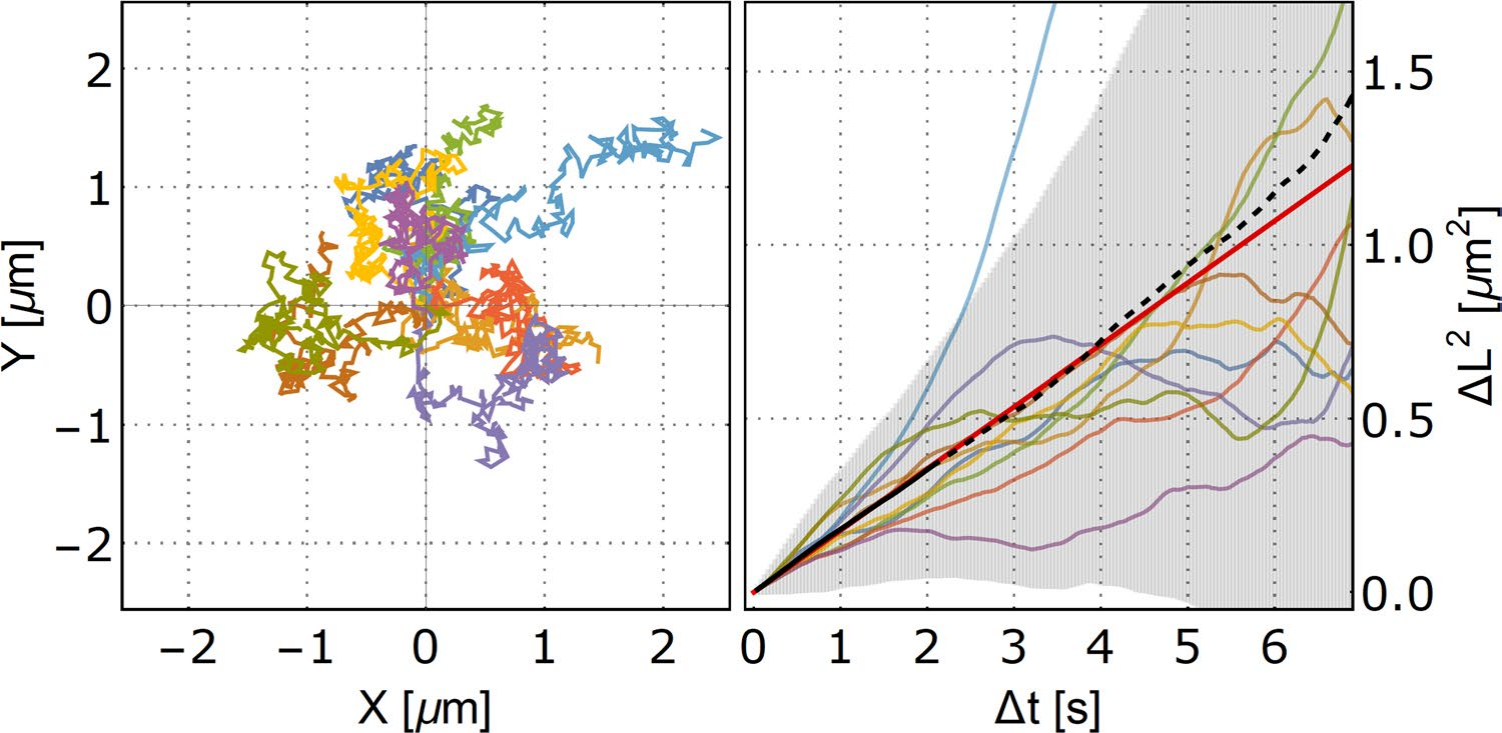
Selected 10 tracks from the simulated ensemble (left) and their corresponding calculated MSD functions (colors match) (right). The thick black line is the combined average of the MSDs (multiple time origins) of the individual particles together with the standard deviation symbolized by the vertical lines. The averaged MSD function is fitted by the formula y = 4DΔ*t*,which yields the diffusion coefficient D.

The mean squared displacements of particle tracks were computed by implementing the known algorithm^13,21^ in Wolfram Mathematica, with the output of the mean $\langle {L}^{2}\rangle$(Δt) and the standard deviation. To average the paths of more particles following the same behavior with multiple time origins, the combined mean MSD at set time delay Δ*t* was computed from gathering the squared displacements Δ*L*^2^ for a set time delay Δ*t* from all tracks and computing the mean and standard deviation of $\langle {L}^{2}\rangle$(Δt). Figure 7 contains the visualization of the result of the MSD algorithm, showing the computed MSDs for a selected ten tracks from the simulated dataset (color lines), together with the averaged MSD over the 10 particle paths (black line) and a linear function fitting the first one-fourth of values of the averaged MSD (red line).

### MSD analysis of particle ensemble diffusion

Figure 7 shows the visualization of the MSD algorithm result of the computed MSDs for 10 selected tracks from the simulated dataset, together with the averaged MSD over the 10 particle paths.

The simplified MSD analysis used in our paper consisted of fitting the MSD values from the first one-fourth of Δ*t* by a linear function $\langle \Delta {L}^{2}\rangle =4D\Delta t$ using a least-squares fit method. The resulting fit yielded the desired value of the diffusion coefficient *D*.

## Supplementary information

Supplementary information
