# Supplementary material for: Nanomotor tracking experiments at the edge of reproducibility
Source: Sci Rep. 2019 Sep 13;9:13222. doi: 10.1038/s41598-019-49527-w (PMC6744498; doi:10.1038/s41598-019-49527-w)
Supplement: Supplementary file 1 — Supplementary information [file 41598_2019_49527_MOESM1_ESM.docx]

Supporting Information

Nanomotor tracking experiments at the edge of reproducibility

Filip Novotný^a^, Martin Pumera^abc*^

^a^Center for Advanced Functional Nanorobots, Dept. of Inorganic Chemistry, Faculty of Chemical Technology, University of Chemistry and Technology in Prague, Technická 5, Prague, Czech Republic

^b^Future Energy and Innovation Laboratory, Central European Institute of Technology, Brno University of Technology, Purkyňova 656/123, Brno, CZ-616 00, Czech Republic

^c^Department of Chemical and Biomolecular Engineering, Yonsei University, 50 Yonsei-ro, Seodaemun-gu, Seoul 03722, Korea

E-mail: martin.pumera@vscht.cz

## Test of the MSD analysis on the simulated particle paths


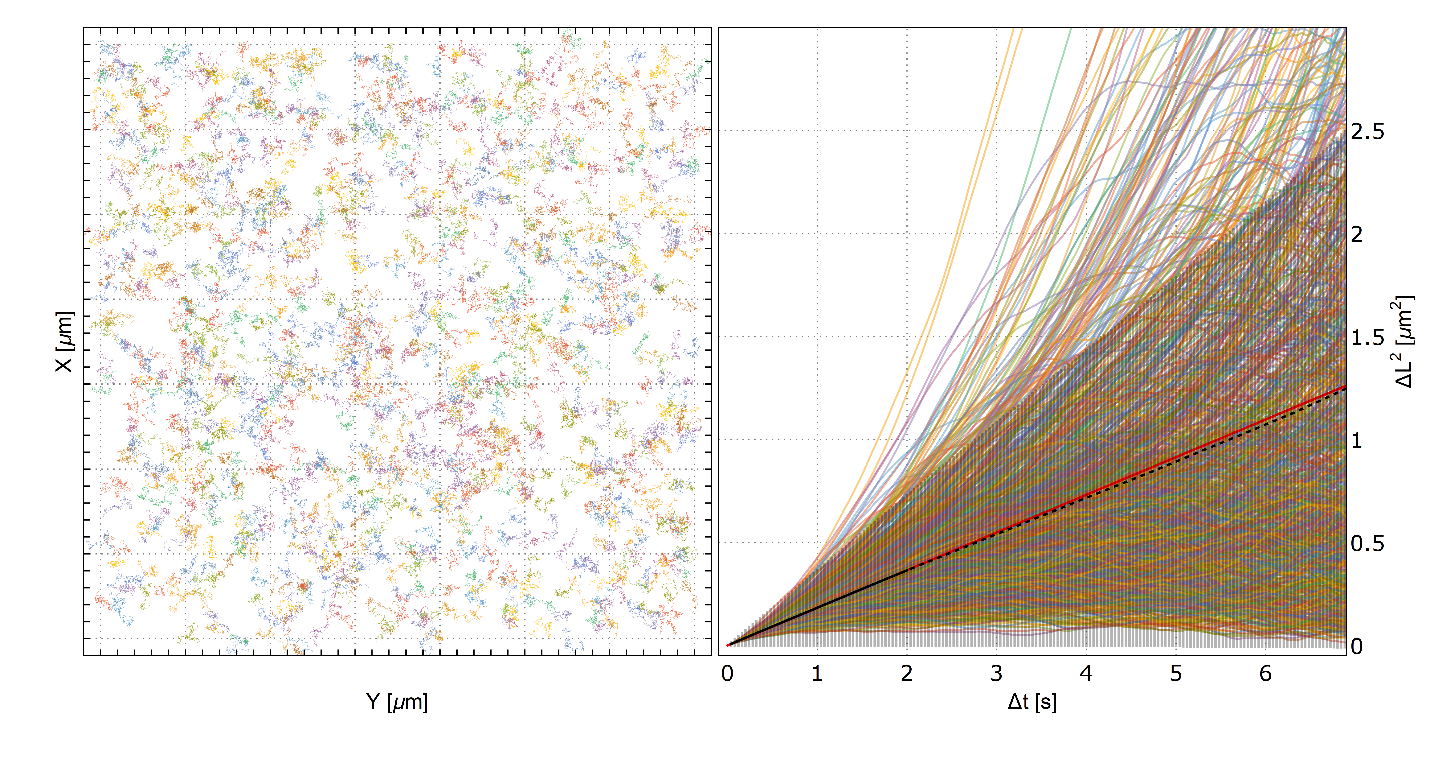


Figure S-1: Visualization and test of the 1000 simulated paths of a 104.3 nm Brownian particle in water. (Left) visualization of all the 1000 particle paths randomly distributed in an XY plane. (Right) A MSD analysis of the whole ensemble of tracks. The colored lines are the MSD plots of each single tracks, the black line is the total averaged MSD over the whole ensemble and the red line is the result of fitting the averaged MSD by a linear function, yielding the mean diffusion coefficient D = 454x10^4^ nm^2^/s.

## *Contour plots of the combination of the number of tracks and the fraction of the total track length used as maximum* $\boldsymbol{\Delta t}$


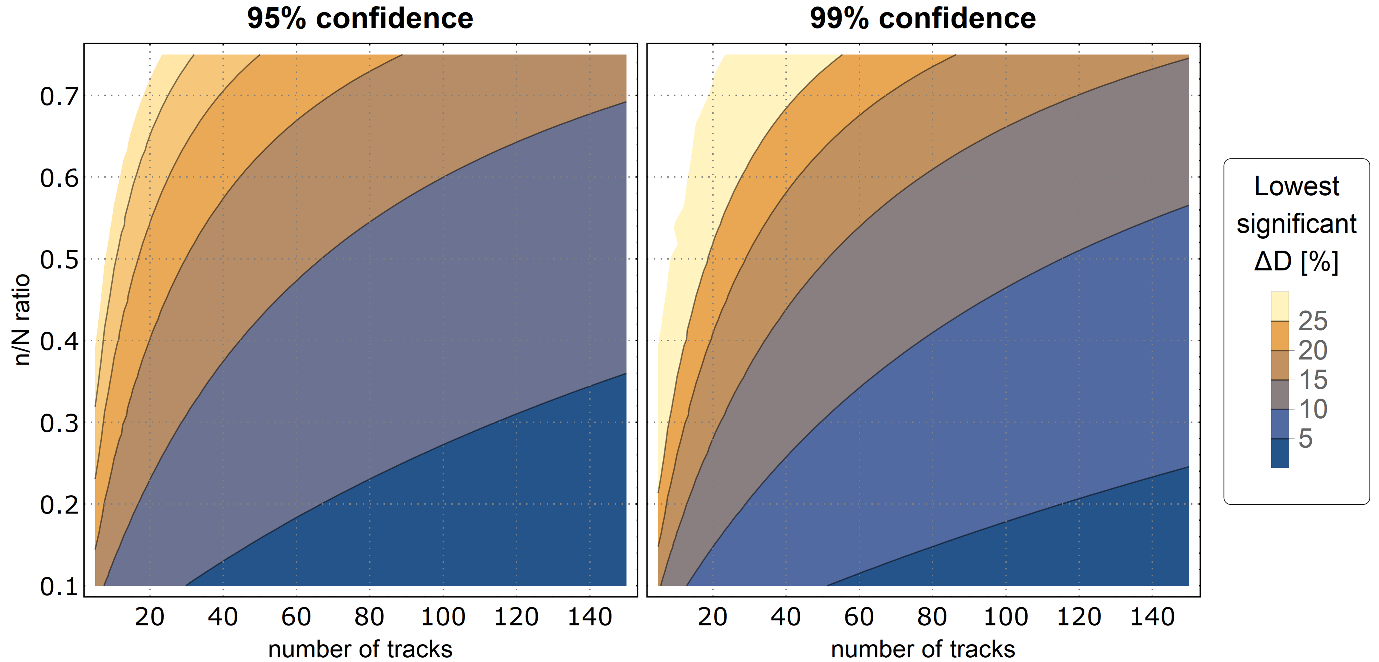


*Figure S-2: Contour plots of the combination of the number of tracks and the fraction of the total track length used as maximum* $\Delta t$ *needed to achieve significance at certain levels of change in the derived diffusion coefficient. Plotted for the 95% and 99% confidence. The plots are meant to give a rough guide to how many tracks of Brownian nanomotors one need to achieve a statistically significant confirmation of a certain amount of observed enhanced diffusion. The values are derived from analytical exploration of single-particle tracking problem for the Brownian movers.*^1^

1. Qian, H., Sheetz, M. P. & Elson, E. L. Single particle tracking. Analysis of diffusion and flow in two-dimensional systems. *Biophys. J.* **60**, 910–921 (1991).
